# Inefficacy or Paradoxical Effect? Uveitis in Ankylosing Spondylitis Treated with Etanercept

**DOI:** 10.1155/2014/471319

**Published:** 2014-06-04

**Authors:** Bernd Raffeiner, Francesca Ometto, Livio Bernardi, Costantino Botsios, Leonardo Punzi

**Affiliations:** ^1^Rheumatology Unit, Department of Medicine, General Hospital of Bolzano, Lorenz Boehler Street No. 5, 39100 Bolzano, Italy; ^2^Rheumatology Unit, Department of Medicine, University of Padova, 35128 Padova, Italy

## Abstract

Ankylosing spondylitis (AS) is presented with axial and peripheral articular involvement. Uveitis is a severe and rather specific manifestation of AS. Biologics targeting tumor necrosis factor (TNF) **α** are effective on both articular and ocular manifestations of disease. The occurrence of uveitis in patients that never had eye involvement or the relapse of uveitis is described during anti-TNF**α** treatment. The frequency of these events is slightly higher during therapy with etanercept. The available TNF**α** blockers show different pharmacokinetics and pharmacodynamics yielding different biological effects. There is an ongoing debate whether uveitis during anti-TNF**α** has to be considered as paradoxical effect or an inadequate response to therapy. Here, we present a case report and review what the evidences for the two hypotheses are.

## 1. Ankylosing Spondylitis: Current Definition and Extra-Articular Manifestations

Ankylosing spondylitis (AS) is the most important among spondyloarthritis (SpA), a family of chronic inflammatory conditions with common epidemiology, immunogenetics, clinics, and radiological features. The group of SpA includes AS, reactive arthritis, psoriatic arthritis, arthritis associated to colitis, and undifferentiated SpA [1]. AS is a chronic progressive inflammatory disease predominantly affecting the axial skeleton and the sacroiliac joints. Enthesitis is the key pathogenetic component of AS and all SpA. Enthesitis is the inflammation of the enthesis, that is, where tendons, ligaments, and joint capsule are attached to the bone. The progressive ossification of enthesis resulting from the chronic inflammation leads to irreversible loss of function [2]. The trigger for the inflammation at this site could be a bacterial antigen and/or other environmental factors that prompt an abnormal immunological activation. In subjects with a predisposing genetic background, this may result in the perpetuation of the immunological response. Interleukine-12 (IL-12), IL-17, and TNF*α* are overexpressed in these patients.

The disease debut is usually around 20 and 40 years, more frequently in male subjects. HLA-B27 gene is often present, while no specific antibodies are found. Signs of systemic inflammation are seldom evident. Diagnosis requires one clinical criterion among chronic inflammatory pain, limited lumbar spine excursion, and limited thoracic expansion and one radiographic criterion, that could be either a bilateral grade II sacroiliitis or a grade III-IV sacroiliitis on one side (according to the 1984 modified New York criteria). In the early stages, magnetic resonance imaging (MRI) has proven to be more sensitive in detecting sacroiliitis, preceding changes on conventional radiography even for many years. In fact, new Assessment of SpA International Society (ASAS) criteria for axial SpA allow the diagnosis of a preradiographic axial SpA in case there is a sacroiliitis on MRI along with one other SpA feature, or HLA-B27 positivity along with two SpA features. In any case, progression to AS is not definite [3].

First line therapy for SpA is physical exercise together with nonsteroidal anti-inflammatory drugs (NSAIDs). Corticosteroids, immunosuppressants, and biologics can be then considered.

SpA present mostly with peripheral arthritis, dactylitis, enthesitis, and, nonetheless, extra-articular manifestations. The burden of these manifestations includes uveitis, psoriasis, inflammation of the pulmonary parenchyma and of the pleura, neuropathies, gastrointestinal inflammation, urogenital involvement, cardiac valvular pathologies, myocardium and conduction tissue dysfunction, renal involvement with microhematuria, IgA-associated glomerulonephritis, and secondary, amyloidosis, generalized, and periarticular osteoporosis [4].

## 2. Clinics, Outcomes, Pathogenesis, and Role of TNF-*α* in Uveitis of Spondyloarthritis 

The most frequent nonarticular manifestation in AS is the inflammatory eye disease. Ninety percent of uveitis is anterior uveitis and is monolateral. Anterior uveitis can be found in up to 30% of AS cases [5]. Anterior uveitis comprehends iritis (i.e., the inflammation of the iris and of the anterior chamber of the eye), iridocyclitis (i.e., inflammation of the iris and of the ciliary body, with inflammatory cells in the aqueous humor), and cyclitis (i.e., inflammation of the ciliary body and of the aqueous humor). In 9.1% of the cases, uveitis is bilateral. Other structures of the eye are seldom involved (3.5%): posterior uveitis (choroiditis or retinochoroiditis), intermediate uveitis (vitritis, peripheral retinitis, and pars planitis), or panuveitis, in case that more than one segment of the eye is involved.

Uveitis is typically acute, with red and painful eye, photophobia, hyperlacrimation, and blurred vision. It resolves in 2 or 3 months and usually has no sequelae. In SpA patients, relapses are frequent (50.6%) often in the other eye. If treatment is not proper, it can cause hypopyon, cataract, glaucoma, synechiae (39.5%), and macular edema (19.7%). Visual loss is therefore a possible outcome (8.3% of uveitis).

Association between uveitis and HLA-B27 positivity is well known. The HLA-B27 positivity correlates with a worse prognosis and frequent relapses. Uveitis incidence correlates linearly with the disease duration up to 20 years and then reaches a plateau. It is also significantly associated with cervical pain, prior diagnosis of inflammatory bowel disease, Short Form-36 (SF36), physical impairment, and disease debut after an infection. Moreover, AS patients with uveitis have worse BASDAI and BASFI scores.

The pathogenesis of the eye disease in AS has not yet been cleared: specific genetical susceptibility, both innate and adaptive immunological systems, and, in the end, an environmental trigger are implicated [6, 7].

The major genetic predisposing factor is HLA-B27, especially the B∗2704 allele and, to a lesser extent, the polymorphisms of LMP2 and HLA-DR8 genes. HLA-B27 positivity is associated with higher TNF*α* levels in active uveitis [8]. Patients who also have A allele in −238 and −308 nucleotides of the TNF*α* gene promoter are more prone to uveitis. Estrogens are proved to inhibit IL-1, IL-6, and TNF*α* expression through nitroxide production. Toll-like receptors (TLR) genes recently appeared to be implicated in the eye disease of AS, together with the killer cell immunoglobulin-like receptors (KIR) and the vitamin-D binding protein. Th17 lymphocytes are implicated in uveitis through the production of IL-17 and IL-23. In animal models deficiency of either interferon-*γ* or IL-1 receptor antagonist leads to higher ocular inflammation. Notwithstanding these evidences, the most important effects are likely due to HLA-B27 positivity and TNF*α* expression.

## 3. Conventional Treatment and TNF*α* Inhibitors in the Treatment of Uveitis

Uveitis has to be regarded as an ophthalmological emergency in order to prevent irreversible outcomes. The choice of treatment must be driven by severity of inflammation and by response to therapy. Topical treatment with corticosteroids is usually effective. Mydriatics and cycloplegics reduce the spasms of the ciliary muscle and thus the pain and, importantly, avoid development of posterior synechiae. About 13–19% of patients do not respond to the topical treatment and inflammation becomes chronic. In these cases, intraocular steroid injections should be preferred to systemic corticosteroids.

Immunosuppressants are sometimes needed in case of involvement of posterior eye structures or in case of frequent relapses and chronic disease.

Sulfasalazine (SSZ) has been shown to reduce frequency and severity of uveitis in case of more than 3 relapses a year [9, 10]. Methotrexate (MTX) lowers significantly the number of uveitis relapses: 0.89 episodes a year with MTX compared to 3.4 episodes without MTX [11]. NSAIDs have a positive effect on recurrence (0.53 episodes a year compared to 2.84); besides, they can be used to control the articular manifestations of AS [12].

In cases of uveitis refractory to immunosuppressants and in case of involvement of posterior eye structures, anti-TNF*α* are recommended and effective [13]. A meta-analysis proved that both infliximab (IFX, monoclonal antibody against TNF*α*) and etanercept (ETA, the soluble receptor of TNF*α*) significantly reduce uveitis recurrences (3.4 and 7.9 per 100 patient-years, respectively, compared to 15.6 of placebo). Adalimumab (ADA, monoclonal antibody against TNF*α*) reduced the number of recurrences by 50% [14]. A retrospective study found that the anti-TNF*α* antibodies, IFX and ADA, were more effective in preventing relapses than the placebo (9.0 versus 47.4 episodes per 100 patients-years for IFX and 0 versus 60.5 for ADA). ETA was not superior to placebo (54.6 versus 58.5 per 100 patients-years) in one study [15]. Further studies proved that ETA is more effective than placebo (8.6 versus 19.3 per 100 patients-years) or at least as effective as SSZ (10.7 versus 14.7 per 100 patient-years) [16]. All anti-TNF*α* improved the treatment of SpA uveitis, particularly, the antibodies, IFX and ADA.

## 4. A Paradoxical Effect: Uveitis during Anti-TNF*α*

Several cases of new onset uveitis or uveitis relapses have been reported during anti-TNF*α* treatment, while the other articular manifestations where controlled by the therapy [17]. Contradictory effects of anti-TNF*α* are also psoriasis-like cutaneous lesions and inflammatory bowel manifestations. A French national surveillance and numerous English case reports highlighted the association between TNF*α* inhibitors and uveitis, with 31 and 121 cases, respectively. ETA was mostly associated with uveitis: 23 of the 31 French uveitis cases and 103 of the 121 English cases had been treated with ETA [18]. There is a difference in the mechanism of action between antibodies and the receptor: besides TNF*α*, ETA inhibits also TNF*β*. In an animal model of uveitis, higher TNF*β* was found, and therefore ETA is expected to be even more effective [19]. Nevertheless, uveitis seems to be specifically associated with ETA and not with all anti-TNF*α* [17].

One theory for ETA-associated uveitis is that, when the soluble receptor ETA binds with TNF*α*, it prevents the clearance of TNF*α* and prolongs its half-life and thus the presence within the eye structures. As is well known, anti-TNF*α* antibodies are effective in Chrohn's disease when compared to ETA. In Chrohn's disease, a defect in T lymphocyte apoptosis is present. ETA does not induce apoptosis and it increases lymphocyte stimulating cytokines, whereas IFX decreases them [20]. The lymphocyte inhibition could explain also the increased frequency of tuberculosis reactivation especially during the therapy with antibodies.

In any case, uveitis is described also during therapy with the anti-TNF*α* antibodies and has also been successfully treated with ETA. Still, uveitis is a manifestation of SpA: assuming uveitis is an adverse event triggered by anti-TNF*α* inhibitors might be an awkward statement [17, 18]. Uveitis can occur long time after the start of anti-TNF*α* therapy, usually after 12–27 months. Some cases had benefit with the switch to another TNF blocker, but in most reported cases uveitis was solved without anti-TNF*α* interruption. Further, in some cases, the reintroduction of the same anti-TNF*α* did not cause disease relapse.

## 5. Case Report

A 19-year-old patient suffering from inflammatory back pain was treated with NSAIDs on demand by her general practitioner. Three years afterwards, she developed polyarthritis and tenosynovitis in the hands, knees, and feet. She was referred to a rheumatologist and she was found to be HLA-B27 positive, systemic inflammation was present, and pelvic radiographs showed bilateral grade 2 sacroiliitis. A diagnosis of AS was then made. The patient was treated successfully with methyl-prednisone 4 mg and SSZ 2000 mg daily. After two years, disease relapsed. MTX at the dose of 15 mg a week did not improve arthritis or inflammation after 6 months of treatment. At the age of 25 years, she started IFX, 200 mg every 6 weeks, in combination with MTX and achieved complete remission (BASDAI score 0 and normal blood exams). After two years, she experienced a severe infusion reaction to IFX with hypotension and dyspnea. She was switched to ETA, 25 mg twice weekly. The patient maintained complete remission for 2 years, and then ETA was tapered to 25 mg weekly. Three years afterwards, she presented with blurred vision, photophobia, hyperemia of the conjunctiva, and pain in the left eye. The ophthalmologist diagnosed an anterior uveitis of the left eye and suggested treatment with topical steroids. As she had no improvement, after one week she was switched to ADA, 40 mg every two weeks and the uveitis recovered promptly. Indeed, the patient started complaining about back pain and peripheral arthritis after a few weeks. Following discussion with the patient and the ophthalmologist, ETA was started at 25 mg twice a week. The patient achieved disease remission shortly after the start of ETA and to date, after 5 years, no other relapses of uveitis or articular disease have been reported.

## 6. Discussion

Inhibitors of TNF*α* are very effective in all SpA and AS manifestations. Monoclonal antibodies appear to be more effective in controlling uveitis compared to the soluble TNF receptor ETA. However, successfully treated uveitis is described with all anti-TNF*α* therapies. Uveitis during TNF*α* inhibitors could be regarded as a paradoxical effect. Likewise, psoriasis is induced by TNF*α* blockers, although these therapies are very effective in the treatment of the skin disease. Presumably TNF*α* blocking may create an imbalance in cellular and cytokine networks disturbing the immunoregulatory function of TNF*α* on autoreactive T-cells, Th17 and T-reg cells [21].

This statement might not be appropriate in our case, as the reintroduction of ETA led to no recurrence of uveitis. Uveitis during TNF*α* inhibitors could rather be the sign of an insufficient control of the disease. Our case proves that IFX adequately blocked the disease manifestation until probably antidrug neutralizing antibodies hampered its efficacy and induced an allergic reaction. ETA then adequately controlled the disease, but, when the dose was lowered, uveitis occurred. Another monoclonal antibody, ADA, had no efficacy on articular manifestations of the disease. In the end, full dose ETA achieved and maintained remission of all disease manifestations for a long time.

Thus, ETA should be deemed as an effective treatment choice in patients with SpA and uveitis, especially in view of the fact that all other therapeutic pathways targeted by newer biologicals seem to be ineffective [22].
